# Radiomics-based Machine Learning Models for Classifying Breast Cancer on Dynamic-Contrast Enhanced MRI through Multi-Observer Analysis

**DOI:** 10.2174/0115734056403426251205212737

**Published:** 2026-03-03

**Authors:** Faikah Awang Ismail, Muhammad Khalis Abdul Karim, Mohd Mustafa Awang Kechik, Izdihar Kamal, Zahurin Ismail, Erliananiza Mohd Salim, Kaltham Abdulwahid Noor

**Affiliations:** 1 School of Biology, Faculty of Applied Sciences, Universiti Teknologi MARA, Cawangan Negeri Sembilan, Kampus Kuala Pilah, 72000, Malaysia; 2 Department of Physics, Faculty of Science, Universiti Putra Malaysia, Selangor, 43400, Malaysia; 3 Radiology Department, Hospital Putrajaya, Ministry of Health Malaysia, 62250, Putrajaya, Malaysia; 4 Radiology Department, Institut Kanser Negara, Ministry of Health Malaysia, Putrajaya, Malaysia; 5 Dubai Health Academic Corporate, Radiology Department. Rashid Hospital, Dubai, UAE

**Keywords:** Breast cancer, Radiomic features, Dynamic Contrast Enhanced, Machine learning (ML), Inter-observer analysis, LASSO regression

## Abstract

**Introduction:**

Breast cancer remains a significant global health issue, necessitating rapid alternative diagnostic methods to improve survival rates. This study aimed to evaluate observer performance in the manual segmentation of Dynamic Contrast-Enhanced MRI (DCE-MRI) images and to assess the effectiveness of radiomic features and machine learning (ML) in classifying benign and malignant breast cancer.

**Methods:**

Breast lesions from 155 patients (65 benign, 90 malignant) were manually segmented on DCE-MRI images by four experienced radiologists using 3D Slicer (version 5.6.1). From each lesion, 107 radiomic features, including shape, first-order, and texture features, were extracted, yielding a high-dimensional dataset. All features were normalized using Z-score scaling. Feature selection was performed using LASSO regression with five-fold cross-validation. The dataset was divided into training and testing sets in a 70:30 ratio, and model performance was evaluated using five-fold cross-validation. The top 20 radiomic features were selected based on intraclass correlation coefficient (ICC) analysis to ensure feature stability. Nine machine learning models, CatBoost, Random Forest, XGBoost, AdaBoost, Naïve Bayes, Logistic Regression, k-NN, SVC, and MLP were employed for classifications. Hyperparameter tuning was applied to optimize model performance, and SHapley Additive exPlanations (SHAP) were used to identify key predictive features.

**Results:**

ICC values ranged from 0.941 to 0.992 (95% CI), demonstrating excellent reliability across all radiomic feature categories. CatBoost outperformed the others with an AUC of 0.937 (95%CI:0.852-0.993) with a sensitivity of 0.889 and a specificity of 0.909 in the internal test set. Other models, such as Random Forest (AUC:0.881, 95%CI:0.758-0.972) and Naïve Bayes (AUC:0.843,95%CI:0.707-0.949), performed well but were less effective compared to CatBoost. SHAP analysis showed that several radiomic features were significant in distinguishing malignant lesions.

**Discussion:**

Ensemble-based models generally outperformed traditional classifiers, such as Logistic Regression and k-NN, possibly because they can capture non-linear relationships in the dataset. SHAP analysis provided insight into model interpretability by identifying key features that contributed most significantly to the classification task.

**Conclusion:**

This study demonstrates the potential of integrating radiomic features with ML for breast cancer classification. CatBoost exhibited the highest predictive performance, highlighting its effectiveness in distinguishing malignant from benign lesions.

## 1 INTRODUCTION

Breast cancer is the most common cancer worldwide, with approximately 2.3 million new cases reported in 2022 and 685,000 related deaths [1]. Southeast Asia is currently facing a growing burden of breast cancer. In 2020, the region recorded approximately 158,939 new cases, corresponding to an incidence rate of 41.2 per 100,000 females. By 2040, the mortality rate is projected to rise to about 15 per 100,000 as the population increases and ages [2]. Among Malaysian women, the incidence of breast cancer has risen, with age-standardized rates increasing from 34.1 per 100,000 during 2012–2016 to 38.9 per 100,000 in 2017–2021 [3]. Approximately 50.5% of cases are detected at later stages (Stage 3 or 4), contributing to higher mortality. This increasing incidence and late-stage diagnosis highlight the urgent need for improved screening and healthcare services to reduce mortality in the country [4, 5]. Mammography demonstrates lower sensitivity than Magnetic Resonance Imaging (MRI), particularly for detecting ductal carcinoma in situ and invasive carcinoma [6]. Dynamic contrast-enhanced MRI (DCE-MRI) has been recognized for its ability to reveal specific morphological and kinetic characteristics of breast lesions, enabling differentiation between benign and malignant cases [7].

Radiomic feature analysis involves extracting quantitative data from medical images through advanced mathematical techniques. These features capture patterns that may not be visually appreciable to the human observer [8]. They include shape descriptors, pixel or voxel intensity values, textural characteristics, and statistical measures that reflect underlying tissue abnormalities. Malignant lesions typically exhibit more irregular shapes and heterogeneous textures compared with benign lesions, often showing sharper contrast enhancement. Such characteristics are captured using radiomic features derived from methods, such as the Gray-Level Co-occurrence Matrix (GLCM), which quantifies texture and shape irregularity [9, 10]. Radiomics also supports data integration across imaging modalities, including MRI, CT, and Positron Emission Tomography (PET), providing a comprehensive view of tumour characteristics. This integration facilitates molecular subtype prediction, treatment planning, and longitudinal monitoring for personalized care [10, 11].

Previous studies reported that models combining radiomic features with pharmacokinetic parameters from DCE-MRI could predict sentinel lymph node metastasis in breast cancer patients, achieving AUC values of 0.80 (95% CI: 0.72–0.89) in the training cohort and 0.74 (95% CI: 0.59–0.90) in the validation cohort [12]. A meta-analysis further demonstrated that combining radiomics with machine learning yielded a pooled sensitivity of 0.81, specificity of 0.85, and an AUC of 0.90 for predicting axillary and sentinel lymph node metastasis, underscoring the robustness of this approach [13]. Diagnostic performance of DCE-MRI radiomics improves when integrated with other modalities, such as PET or additional MRI sequences, such as diffusion-weighted imaging (DWI). The AUCincreased to0.983(95% CI: 0.962–1.000) with multi-modal integration compared with 0.889 (95% CI: 0.841–0.937) using DCE radiomics alone [14]. Similar findings were also reported in previous studies, with AUC values rising from 0.82 for DCE alone to 0.89 for DCE combined with DWI, and up to 0.92 when T1-weighted, T2-weighted, DWI, and DCE sequences were integrated [15]. Such accuracy is essential for reducing unnecessary biopsies and ensuring timely treatment for malignant lesions. Therefore, radiomic features extracted from the dynamic properties of DCE-MRI can support the development of models for evaluating tumour behaviour and treatment response [16].

Recent advances in artificial intelligence (AI), particularly deep learning (DL), have significantly influenced diagnostic applications in breast imaging. Convolutional neural networks (CNNs) have demonstrated the ability to detect malignancies up to a year earlier than radiologists [17]. As AI becomes increasingly embedded in clinical workflows, explainability and transparency have become critical considerations. Ghasemi *et al*. [18] emphasized the growing importance of explainable AI (XAI) in breast cancer diagnostics, identifying Shapley Additive exPlanations (SHAP) as a widely used method for interpreting model decisions in both diagnostic and prognostic settings. Complementing this, Hirsh *et al*. [17] proposed a comprehensive machine learning and deep learning framework for early breast cancer detection on MRI, integrating tumour segmentation, radiomic feature extraction, and classification. Their pipeline demonstrated strong performance and highlighted the feasibility of automating complex imaging workflows. Additional studies have reported similar findings, including multimodal MRI models that integrate T1-weighted and T2-weighted imaging with clinical metadata using attention-based fusion strategies to enhance classification performance [19]. Kim and Park [20] further proposed a radiomics-guided multimodal attention network combining DCE-MRI and Apparent Diffusion Coefficient (ADC) maps to predict pathological complete response (pCR), demonstrating the added value of incorporating handcrafted radiomic features within deep learning frameworks for treatment monitoring.

Similarly, Yang *et al*. [21] implemented a hybrid deep learning pipeline that integrated handcrafted radiomic features with CNN-extracted features, achieving higher diagnostic accuracy than standalone models. However, the choice of AI technique must be guided by data characteristics. Prinzi *et al*. [22] compared shallow and deep learning classifiers and found that shallow machine learning often yields equal or superior performance, particularly in small- to medium-sized, well-structured datasets using handcrafted radiomic features. Given our relatively small sample size (n = 155) and reliance on semantically rich radiomic features, we selected a shallow machine learning method due to its greater interpretability, faster training time, and minimal computational requirements. These factors are especially advantageous in clinical environments where data analysts and high-performance computing resources may be limited.

Despite the strong potential of radiomics in breast cancer diagnosis, its performance is influenced by the observer’s expertise during the segmentation process. Experienced observers are better equipped to differentiate benign from malignant lesions, improving segmentation quality and diagnostic performance [23]. Therefore, this study aimed to evaluate the performance of radiologists in manually segmenting breast lesions on DCE-MRI and to assess the effectiveness of radiomic features combined with machine learning models for classifying lesions as benign or malignant. We hypothesized that (i) manual segmentation would yield high inter-observer reliability, (ii) radiomic features derived from DCE-MRI would accurately differentiate benign from malignant lesions, and (iii) ensemble-based models, such as CatBoost, would outperform conventional machine learning methods in classification tasks.

## 2 MATERIALS AND METHODS

### 2.1 Patient Selection

**Fig. (1):**
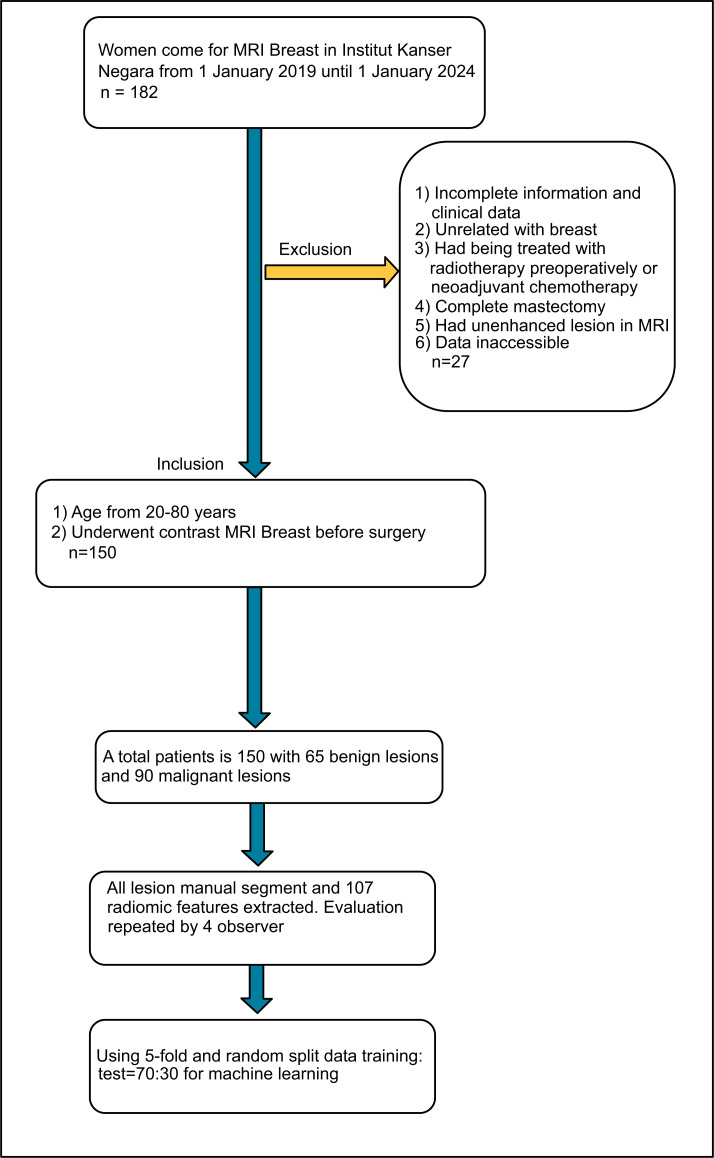
Patient inclusion and exclusion criteria.

A retrospective radiomics study was conducted at Institut Kanser Negara, Putrajaya, where breast MRI data from patients were reviewed from January 1^st^, 2019, to January 1^st^, 2024. The study was approved by the Medical Research & Ethics Committee, Ministry of Health, on January 15^th^, 2024 (Ethics Number: NMRR ID-23-03516-NMY (IIR)), and patient consent was waived. The inclusion criteria were: (1) women aged 20 to 80 years, (2) having undergone contrast-enhanced breast MRI prior to surgery, and (3) having a complete MRI breast protocol with good-quality images. The exclusion criteria were: (1) incomplete clinical information, (2) poor-quality MRI breast images, (3) prior preoperative radiotherapy or neoadjuvant chemotherapy, (4) complete mastectomy, (5) unenhanced lesions on MRI, and (6) inaccessible data due to hardware or software issues. Lesion classification (benign or malignant) was confirmed through histopathological examination documented in the patients’ clinical records and served as the ground truth for training and evaluating the machine learning models. (Figure **1**) illustrates the patient inclusion and exclusion process. As this was a retrospective study, the sample size was not predetermined through power analysis. Instead, it was based on the total number of eligible cases that met the inclusion and exclusion criteria within the study period, resulting in a final sample of 155 patients out of 182 reviewed records. This approach was consistent with the methodology used by Granzier *et al*. [24] and Debbi *et al*. [25], both of which also employed retrospective, single-center designs with manual segmentation and radiomics-focused analyses.

### 2.2 MRI Acquisition Protocol

Images were acquired using a 3-Tesla MRI system (Magnetom Vario, Siemens, Erlangen, Germany) equipped with a 16-channel breast coil. Each examination included several imaging protocols: T1-weighted: TR 6 ms, TE 2.46 ms, slice thickness 1.5 mm, FOV 480 × 480 mm, flip angle 15º. T2-weighted: TR 7370 ms, TE 71 ms, slice thickness 4.0 mm, FOV 480 × 480 mm, flip angle 15º. Diffusion-weighted imaging (DWI): TR 6000 ms, TE 79 ms, slice thickness 4 mm, FOV 480 × 480 mm, flip angle 90º. Dynamic contrast-enhanced (DCE) MRI: TR 4.54 ms, TE 1.73 ms, slice thickness 1.5 mm, FOV 480 × 480 mm, flip angle 10º. For the DCE protocol, one pre-contrast phase and five post-contrast phases were acquired, with a 30-second interval between phases. Five subtraction image sets were generated. Gadobutrol was administered at a dose of 1.0 mmol/ml/kg and an injection rate of 2.0 ml/s, followed by a 20 ml saline flush.

### 2.3 Lesion Segmentation

Breast lesion segmentation was performed manually by a team of four experienced radiologists using 3D Slicer software (version 5.6.1; https://www.slicer.org). The team comprised two senior breast radiologists with 15 years of experience and two radiologists with 8 years of experience. The region of interest (ROI) was delineated on the pre-processed images prior to radiomic feature extraction, as shown in Fig. (**2**).

In cases where multiple lesions were present in the breast, only the largest was selected for analysis. However, if both benign and malignant lesions were present in the same breast, each lesion was segmented according to its respective classification. The lesion segmentation includes all lesions within the breast area. A total of 107 radiomic features were extracted, categorized as follows: 14 shape features, 18 first-order features, and 75 texture features. The texture features included 24 from the Gray Level Co-occurrence Matrix (GLCM), 14 from the Gray Level Dependence Matrix (GLDM), 16 from the Gray Level Run Length Matrix (GLRLM), 16 from the Gray Level Zone Size Matrix (GLSZM), and 5 from the Neighboring Gray Tone Difference Matrix (NGTDM).

Since the performance of every observer was evaluated, an independent segmentation method was applied. To ensure uniformity in the output, a standard bin width of 25 was set before feature extraction. The value of radiomic features was normalized using Z-score and Pearson correlation to map the values between 0 and 1 prior to heatmap analysis. Z-score scaling was applied to the radiomic data using the fit_transform function from the Python scikit-learn library prior to machine learning (ML) model construction. The Z-score can be defined as Eq. (**1)**:

**Table:** 

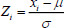	(1)

Where *x_i_* is the original radiomic feature value, *µ* is the mean of the feature, and σ is the standard deviation of the feature. To reduce the severity of data processing, only features with p > 0.05 in the ICC were selected. The Least Absolute Shrinkage and Selection Operator (LASSO) regression model was also used in the feature selection process, incorporating 5-fold cross-validation. Mathematically, LASSO can be defined as Eq. (**2)**:

**Table:** 

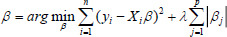	(2)

Where *β* is the value of the regression coefficient, *y_i_* is a target variable, *X_i_* is a feature vector, and *λ* is a regularization parameter. This model will help reduce the number and redundancy of radiomic features.

### 2.4 Machine Learning (ML) Models

This study employed shallow machine learning (ML) methods due to their suitability for clinical settings, fast processing times, and minimal computational requirements. Nine ML models were developed to identify radiomic features that effectively differentiate benign from malignant breast lesions. The models included CatBoost (Categorical Boosting), Random Forest, XGBoost (Extreme Gradient Boosting), AdaBoost (Adaptive Boosting), Naïve Bayes, Logistic Regression, k-Nearest Neighbors (k-NN), Support Vector Classifier (SVC), and Multi-layer Perceptron (MLP). The top 20 radiomic features capable of distinguishing benign from malignant lesions were selected based on the intraclass correlation coefficient (ICC) results. These features were initially evaluated using the test set, followed by model training. Hyperparameter tuning was conducted to optimize model performance. SHAP analysis was then applied to the best-performing model to assess the contribution of individual radiomic features in differentiating the lesions. This can be defined as Eq. (**3)**:

**Table:** 

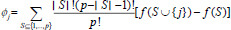	(3)

Where *φ_j_* represents the SHAP value of feature *j*, *S* is a subset that excludes *j,* and *f*(*S*) is a model prediction using subset S.

### 2.5 Statistical Analysis

All statistical analyses were conducted using SPSS version 28, with a significance level set at p < 0.05. The Shapiro-Wilk test was used for data that were not normally distributed. Categorical data were assessed using the chi-square test. The performance and agreement among observers were evaluated using an ICC reliability test, as defined in Eq. (**4)**.

**Table:** 

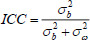	(4)

Where σ*_b_*^2^ is the variance between observers, and σ*_ω_*^2^ is a variance within observers. All quantitative radiomic features were treated as continuous variables. Intraclass correlation coefficient (ICC) values below 0.5 indicate poor reliability, values between 0.5 and 0.75 represent moderate reliability, values from 0.75 to 0.90 indicate good reliability, and values above 0.90 signify excellent reliability [26-28]. Pearson correlation analysis was performed on the top 20 features that distinguished benign from malignant lesions prior to generating the clustered heatmap. For the machine learning (ML) models, performance was evaluated using accuracy, sensitivity, specificity, F1-score, receiver operating characteristic (ROC) curves, and area under the curve (AUC) with 95% confidence intervals (95% CI). All performance metrics were calculated using the Python scikit-learn library.

## 3 RESULTS

### 3.1 Radiomics and Interobserver Stability

A total of 155 out of 185 patients met the eligibility criteria for inclusion in this study. Table **1** presents the demographic characteristics of the patients, including tumour type and lesion measurements. The dataset was randomly divided into a training set of 110 patients (45 benign and 65 malignant lesions) with a mean age of 41.9 ± 7.9 years, and a test set of 45 patients (20 benign and 25 malignant lesions) with a mean age of 40.6 ± 4.0 years. All variables showed statistically significant differences, except for age and Maximum3DDiameter in malignant lesions within the test set.

While radiologists in this study did not provide diagnostic decisions, their performance in lesion segmentation was assessed through ICC analysis. Table **2** displays the ICC values among interobservers for the selection of radiomic feature categories. The ICC values ranged from 0.941 to 0.992, indicating excellent reliability. Each observer demonstrated significant consistency in ICC values, with Observer 3 (O_3) and Observer 4 (O_4) showing the highest ICC values, particularly in the First Order and GLCM categories, with reliability as high as 0.990 and 0.992, respectively. Observers 1 (O_1) and 2 (O_2) also performed well with ICC values around 0.980 for most categories. The observers’ performance gap was minimal, indicating strong radiomic data interpretation agreement. In the GLRLM feature interpretation, ICC values dropped marginally, with Observer 1 having the lowest ICC of 0.941 across all categories and observers. The slight performance difference among observers was due to their individual experience; both Observers 3 and 4 were radiologists with 15 years of experience.

**Fig. (3):**
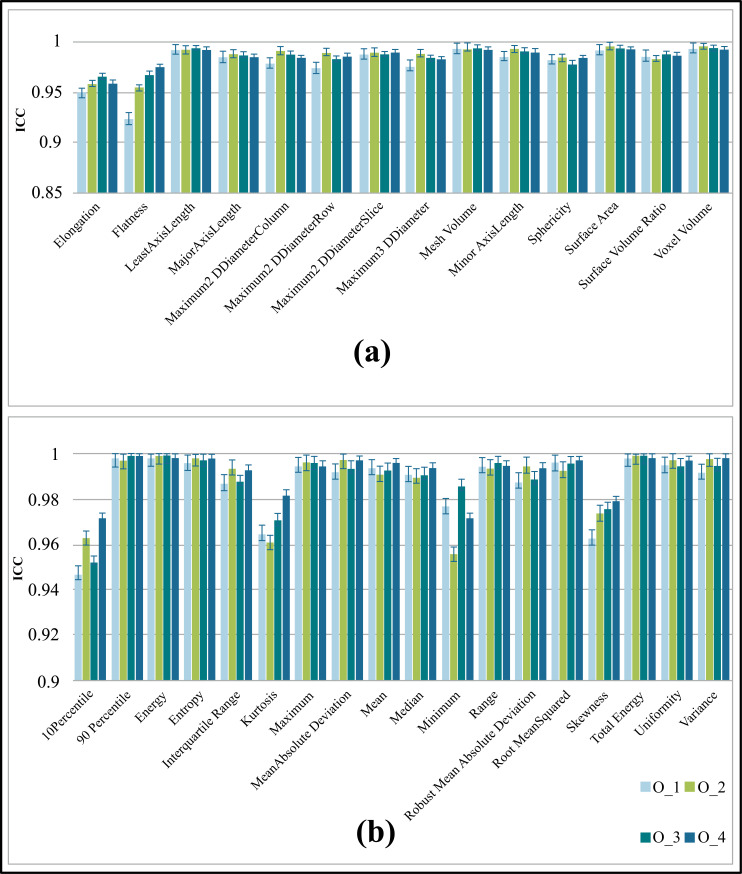
ICC value for (**a**) Shape Features and (**b**). First Order Features.

Figures (**3a**, **b** and **4**) display radiomic features along the x-axis, grouped according to their respective categories, while the y-axis represents the ICC values. (Figure **3**) illustrates the ICC values for both shape and first-order features. Both categories demonstrate high stability, with ICC values ranging from 0.95 to 0.99, indicating strong agreement. Among the shape features, LeastAxisLength, MeshVolume, SurfaceArea, and VoxelVolume show the highest agreement, approaching 0.99 and reflecting near-perfect reliability. Elongation and Flatness present slightly lower ICC values around 0.95, yet still exhibit strong interobserver reliability. Overall, the shape features show excellent consistency across observers, supporting their reliability in differentiating benign from malignant lesions during visual assessment.

For the first-order features, the observers also demonstrated high agreement, with most ICC values exceeding 0.98. 90Percentile, Energy, and TotalEnergy showed the strongest consistency, as illustrated in Fig. (**3b**). In contrast, 10Percentile, Kurtosis, Minimum, and Skewness had slightly lower ICC values, closer to 0.96, indicating reduced but still acceptable consistency.

The GLCM features demonstrated high reliability, with most ICC values exceeding 0.98. The features Contrast, DifferenceAverage, Id, and Idm showed the highest agreement, with ICC values approaching 0.99. Imc1, Imc2, and MCC exhibited slightly lower consistency, just below 0.98. The GLDM features displayed strong agreement as well, with most ICC values ranging from 0.95 to 0.98. DependenceEntropy and DependenceNonUniformity were the most consistent across observers. For the GLRLM features, ICC values ranged from 0.92 to 0.99, indicating overall high agreement. RunEntropy and RunLengthNonUniformity showed the highest consistency (0.96–0.99), whereas LowGrayLevelRunEmphasis and ShortRunLowGrayLevelEmphasis had lower ICC values, averaging between 0.89 and 0.97.

**Fig. (4):**
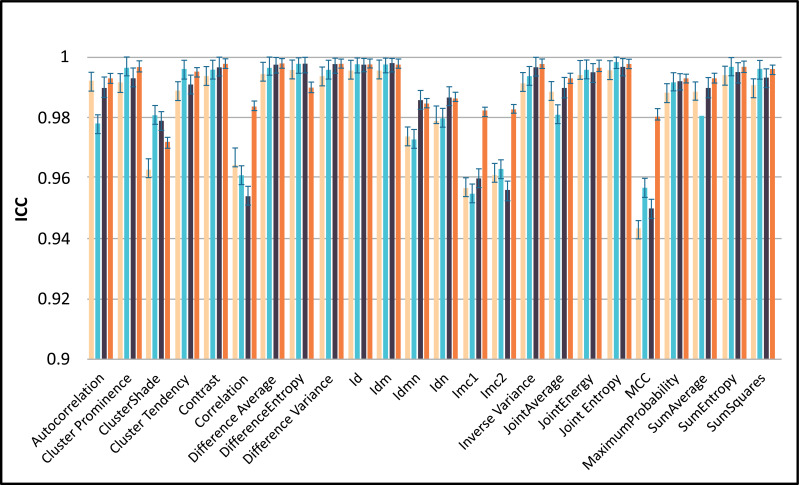
ICC value for selected textural features.

Figure **4** shows the ICC values for selected textural features. GLSZM features exhibited greater variability compared to other texture features. For example, GrayLevelNonUniformity demonstrated high agreement, with ICC values above 0.95, whereas features, such as SizeZoneUniformityNormalized and SmallAreaEmphasis, had lower ICC values around 0.90, indicating reduced consistency. NGTDM features showed moderate to high reliability, with ICC values ranging from 0.90 to 0.99. Complexity exhibited higher consistency, while Strength had slightly lower reliability. Overall, interobserver agreement was high across most texture features, with ICC values exceeding 0.90. However, the variability observed in certain GLSZM and NGTDM features suggests the potential need for further refinement or additional training to enhance observer consistency.

### 3.2 Performance of ML Models

Prior to the ML performance analysis, LASSO feature selection was applied to reduce the number of features while preserving the model’s predictive power. Figure (**5**) presents both the heatmap and SHAP analyses of classification performance. Some selected texture-related features, including original_ glrlm_LongRunLowGrayLevelEmphasis, original_glszm_Sma llAreaLowGrayLevelEmphasis, and original_gldm_Low GrayLevelEmphasis, exhibit significant positive associations, as shown in Fig. (**5a**). This clustering indicates that these features share similar tumor texture properties and may be redundant. The results suggest that removing strongly correlated features could enhance model efficiency without compromising performance. In contrast, original_glcm_MCC and original_glrlm_LowGrayLevelEmphasis show weaker correlations with other features. These less-correlated variables may provide unique information, making them valuable for distinguishing between benign and malignant lesions. To further investigate radiomic features capable of differentiating these lesions, ML models were subsequently built, and their performance was evaluated.

SHAP summary plots illustrate the importance and impact of radiomic features on the CatBoost model’s output for differentiating benign and malignant breast cancer, as shown in Fig. (**5b**). The feature with the highest mean absolute SHAP value is original_gldm_LowGrayLevelEmphasis, which contributes substantially to the model’s decision-making. Other features display notable trends: original_glcm_Imc1 shows higher values correlated with a reduced likelihood of malignancy, suggesting that more homogeneous textures may indicate benign lesions. Conversely, original_glszm_SmallAreaLowGrayLevelEmphasis positively influences malignancy predictions, where higher values, indicating a greater presence of small areas with low gray levels, are associated with increased malignancy risk. Additionally, original_firstorder_10Percentile and original_shape_Maximum2DDiameterRow also have a substantial positive impact on malignancy predictions. In contrast, benign lesions generally exhibit a more stable structure with uniform and regular gray levels in the images [29].

Figure (**6**) illustrates the ROC curves of nine ML models, while Table **3** compares their performance in differentiating benign and malignant lesions within this study’s dataset. The CatBoost model achieves the highest AUC score of 0.998 in the training set, followed by Random Forest, XGBoost, and AdaBoost, with AUCs of 0.987, 0.960, and 0.960, respectively. These ensemble models demonstrate strong discriminatory ability, making them particularly suitable for this classification task. Logistic Regression, k-NN, and Naïve Bayes show moderate performance, with AUC values of 0.950, 0.916, and 0.913, respectively, indicating they are less accurate than the ensemble models. SVC and MLP models perform the least effectively, with AUC values of 0.770 and 0.688, respectively, suggesting lower efficiency for this dataset.

The CatBoost model surpassed other models with an AUC of 0.937, demonstrating an excellent balance between sensitivity (0.889) and specificity (0.909), thus establishing it as the most accurate model for differentiating between benign and malignant lesions. The results indicated that Random Forest achieved an AUC of 0.881; however, its sensitivity decreased to 0.778, and its overall accuracy was 80%, signifying inferior performance compared to CatBoost. Conversely, Naïve Bayes, Logistic Regression, and XGBoost had the lowest AUC scores. XGBoost exhibited difficulty in detecting cancerous lesions, as indicated by its low sensitivity (0.222).

## 4 DISCUSSION

In Malaysia, one in 19 women is diagnosed with breast cancer, underscoring the need for effective patient management and accurate diagnostic technologies [30]. However, factors such as limited awareness, fear of outcomes, and delays in receiving results contribute to late-stage diagnoses, with many women presenting with advanced cancer and poorer prognoses [31]. Such delayed presentation complicates the distinction between benign and malignant tumors, as advanced-stage cancers often exhibit more aggressive characteristics. This challenge can be addressed by enhancing radiologists’ ability to differentiate benign from malignant breast lesions through the use of radiomic features specifically tailored for the Malaysian population. Accordingly, this study aims to evaluate interobserver performance in the manual segmentation of DCE-MRI images using ICC and to assess the effectiveness of radiomic features in distinguishing benign from malignant breast cancer.

The manually segmented radiomic features extracted from DCE-MRI images demonstrated high interobserver reliability in distinguishing benign from malignant breast cancer, with ICC values ranging from 0.941 to 0.992 across all feature categories. Experienced radiologists (Observers 3 and 4) showed the highest ICC values, particularly in the First Order and GLCM categories (0.990 and 0.992), highlighting the robustness of the segmentation process. Minor variations in performance were attributed to differences in observer experience. The reliability of these segmentations is critical for ensuring that extracted radiomic features are accurate and suitable for ML applications. Previous studies have shown that ROI placement affects variability, and utilizing the entire lesion can improve ICC values in DCE-MRI analyses [32, 33]. Standardization of segmentation procedures can further enhance reproducibility and reduce the impact of individual observer experience [29, 34, 35]. While automated segmentation methods are fast and efficient, manual segmentation was preferred in this study for its precision, adaptability to individual patient characteristics, and ability to reduce variability factors essential for accurately distinguishing benign from malignant lesions [10]. High interobserver reliability in manual segmentation enables robust radiomic data generation, which, when combined with ML, can mitigate residual variability due to differences in radiologist experience [23, 36].

Among the ML models evaluated, CatBoost achieved the highest performance in differentiating benign and malignant breast lesions, with an AUC of 0.937 on the test set, along with high sensitivity (0.889) and specificity (0.909). CatBoost excels at handling heterogeneous and categorical data and has demonstrated strong performance across multidisciplinary applications [37, 38]. Random Forest and Naïve Bayes also performed well but exhibited lower AUC and sensitivity values. Several studies have compared ensemble methods to conventional ML techniques [39, 40], showing that ensemble approaches such as CatBoost, Random Forest, XGBoost, and AdaBoost can outperform traditional models like Decision Tree and SVM [41]. Unlike conventional ML, ensemble methods combine multiple models during training, reducing bias and improving processing efficiency. Previous research comparing SVM and ensemble methods, including AdaBoost, Gradient Boost, XGBoost, and LightGBM, found that XGBoost and LightGBM achieved higher AUCs (0.95) compared to SVM (0.88), reinforcing the superiority of ensemble approaches in breast lesion classification [39].

SHAP analysis identified original_gldm_LowGrayLevel Emphasis as the most predictive radiomic feature for breast cancer malignancy, reflecting the heterogeneous texture of malignant tissue. This feature emphasizes lower gray-level intensities, which are indicative of texture patterns associated with malignancy, such as necrotic regions, irregular cellular structures, and heterogeneous tissue density [42]. Benign lesions, including fibroadenomas or cysts, are typically more homogeneous with well-circumscribed boundaries and consistent pixel intensities [43]. Additional predictive features included original_glszm_SmallArea LowGrayLevelEmphasis and original_glcm_Imc1, the latter of which correlated with benign lesions, consistent with their more uniform cell arrangement and smooth boundaries [29]. Malignant tumors often contain multiple small, low-intensity regions corresponding to necrosis, poor perfusion, or dense cellular regions, resulting in heterogeneous textures [7]. Other features, such as original_firstorder_10Percentile and original_shape_Maximum2DDiameterRow, also contributed to malignancy prediction, as lower intensity values and larger tumor sizes are associated with malignancy [25, 44].

Although this study employed shallow ML models on handcrafted radiomic features due to their interpretability and reproducibility, recent advances highlight the potential of deep radiomics and hybrid learning frameworks for superior performance on larger datasets. For instance, Yang *et al*. [21] proposed a Radiomics-Embedded Vision Transformer (RE-ViT), integrating radiomic descriptors into ViT architectures and outperforming CNNs and existing hybrid models in medical imaging tasks. Similarly, Jahan *et al*. [45] developed a hybrid CNN-Vision Transformer for breast cancer patch classification and subtype identification, achieving high diagnostic accuracy. Zhang *et al*. [46] demonstrated that a DCE-MRI-based Vision Transformer could noninvasively predict HER2 expression levels (AUCs 0.80–0.86) across external cohorts, supporting the utility of transformer-based architectures in conventional DCE-MRI applications. For segmentation, Li *et al*. [47] introduced HCMA-UNet, a hybrid CNN-Mamba-Attention network designed for DCE-MRI lesion segmentation, achieving high accuracy with improved computational efficiency. These studies underscore the value of combining local feature extraction (CNN), global attention mechanisms (Transformer), and explainable radiomics to develop robust and generalizable models. While deep learning approaches often require large annotated datasets and complex computational resources, future iterations of this work could incorporate hybrid frameworks as datasets expand.

Overall, this study highlights the potential of DCE-MRI radiomic features combined with ML models to improve breast cancer diagnosis, particularly in differentiating benign from malignant lesions. CatBoost, with high AUC, sensitivity, and specificity, demonstrates the clinical feasibility of these tools. The findings also provide insight into integrating ML into breast cancer screening workflows, enabling more precise clinical decision-making, early detection, and reduced diagnostic errors. Importantly, this work emphasizes the potential for personalized diagnostic tools tailored to the Malaysian population, enhancing the relevance and impact of radiomics-based approaches in local clinical practice.

## 5 STUDY LIMITATIONS

This study has several limitations. First, data were obtained from a single center, which limits the generalizability of the findings. External validation across multiple institutions would enhance the relevance of the results, making them more applicable to diverse populations, MRI machines, and clinical settings. The relatively small sample size also constrains the statistical power of the study and increases the risk of overfitting in ML models. Manual segmentation is labor-intensive and time-consuming for observers; therefore, semi-automated or fully automated segmentation approaches could be explored in future studies.

We also acknowledge the growing interest in deep learning and hybrid models that integrate radiomics with advanced architectures, including CNN-based feature extractors and Vision Transformers, which have demonstrated improved performance in classification tasks, particularly with larger and more variable datasets [21, 45, 47, 48]. However, given the size of our dataset and the emphasis on feature transparency and reproducibility, shallow ML models were preferred for this study. Future research could investigate the integration of deep features or hybrid architectures to enhance classification performance while maintaining interpretability through radiomic profiling.

This study did not include diagnostic classifications by radiologists, as the primary objective was to evaluate interob-server segmentation reliability and assess the performance of radiomics-based ML models using histopathological results as the ground truth. Although incorporating radiologist assessments could increase clinical relevance, it was beyond the scope of the current study. Future work will aim to directly compare radiomic model outputs with radiologist diagnoses to further validate model performance in clinical settings.

## CONCLUSION

In conclusion, this study demonstrates high interobserver reliability in manual segmentation, highlighting the robustness and consistency of the radiomic feature extraction process. Furthermore, it confirms the effectiveness of combining DCE-MRI-derived radiomic features with ML models for breast cancer classification. Among the models evaluated, CatBoost achieved the highest predictive performance, underscoring its capability to accurately distinguish malignant from benign lesions.

## Figures and Tables

**Fig. (2):**
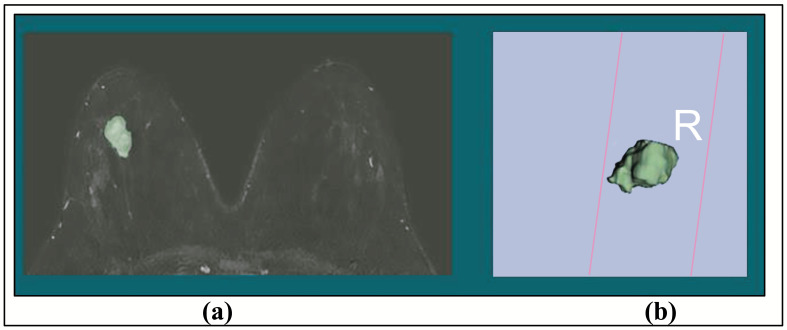
Lesion segmentation, (**a**) Placement of ROI in the image and (**b**) Lesion extracted from the image.

**Fig. (5):**
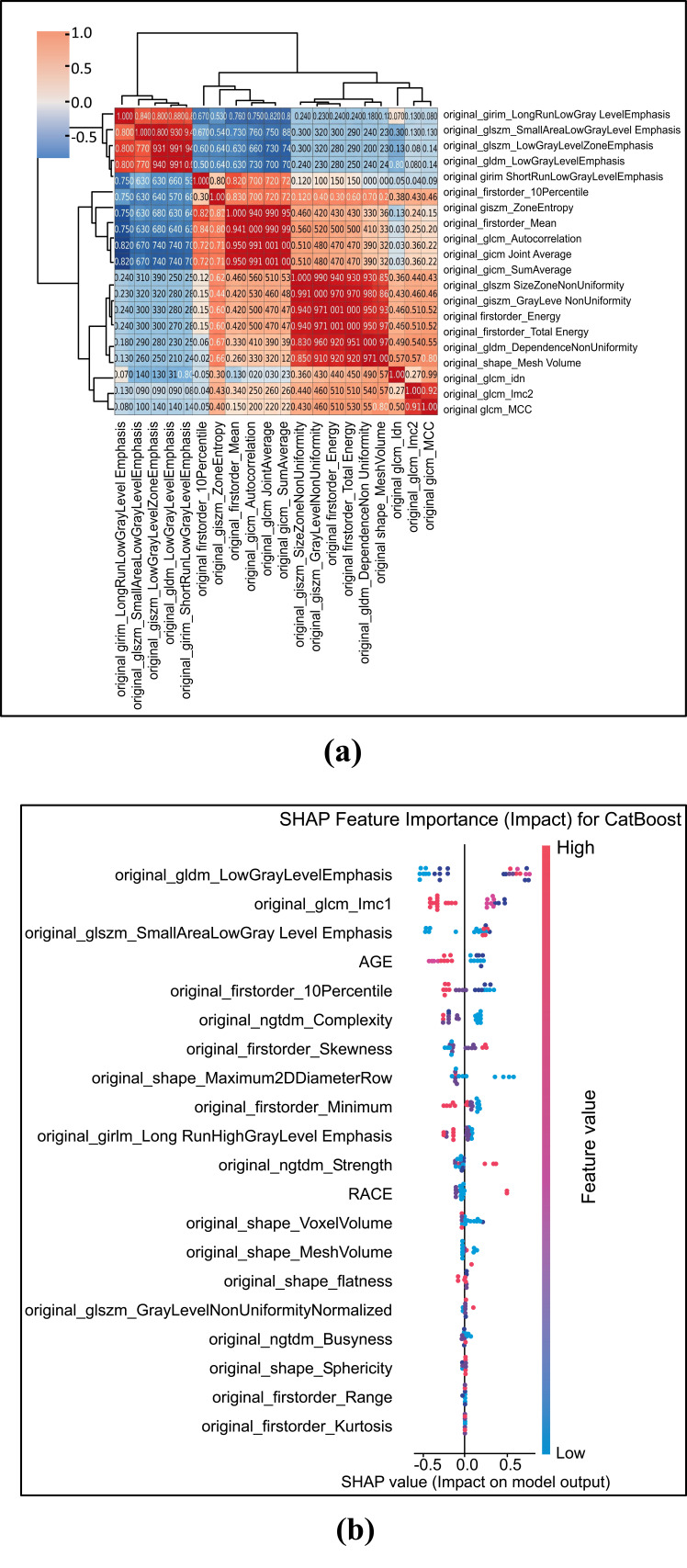
(**a**) Heatmap analysis for the top 20 features in distinguishing benign and malignant lesions, (**b**) SHAP feature importance for CatBoost.

**Fig. (6):**
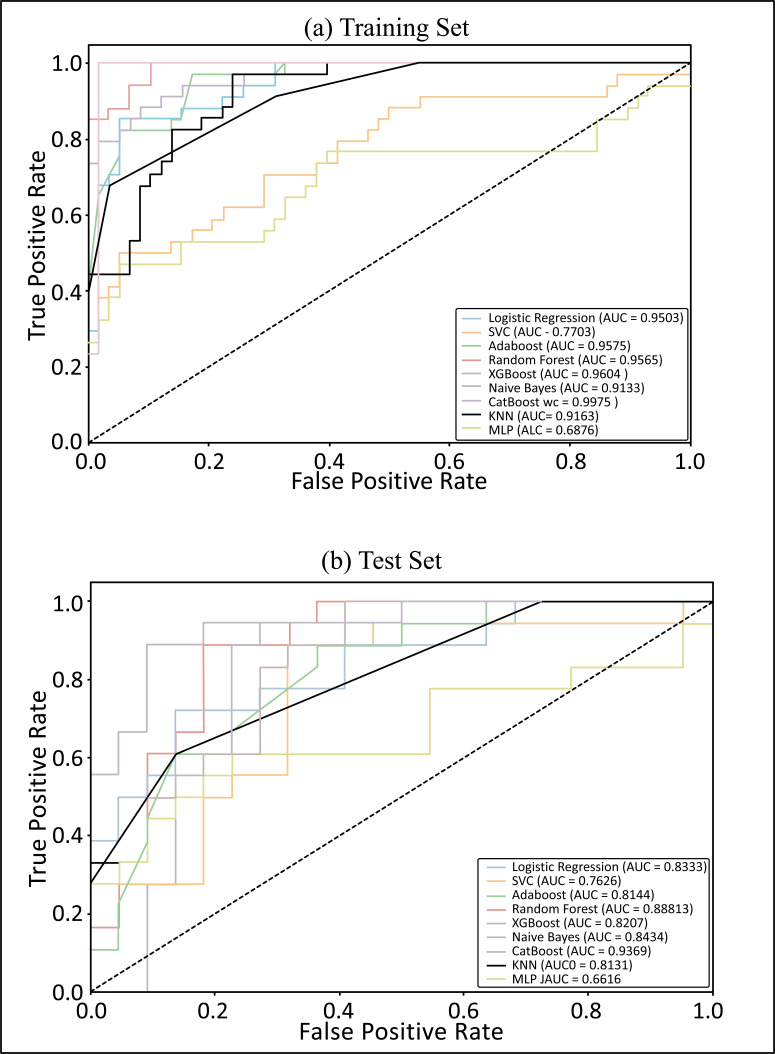
Graph for ROC curve, (**a**) Training set, (**b**)Test set.

**Table 1:** Characteristics of patients and lesions.

**Characteristic**	**Training (n=110)**				**Test (n=45)**			
	**All** **(n=110)**	**Benign** **(n=45)**	**Malignant** **(n=65)**	***p*-value**	**All** **(n=45)**	**Benign** **(n=20)**	**Malignant** **(n=25)**	***p*-value**
Age (years)	41.9 ± 7.9	40.7 ± 6.3	42.1 ± 7.9	0.095	40.6 ± 4.0	41.6 ± 3.0	41.0 ± 4.3	0.51
Maximum3DDiamter (mm)	29.5±15.9	19.5±8.0	35.9±16.6	<0.05	35.9±16.6	43.1±28.6	27.1±11.4	0.22
SurfaceArea (mm)	2177.0±2835.3	892.4±808.3	3002.8±3365.3	<0.05	3377.8±4106.0	5665.9±5869.0	1852.3±1643.0	0.06
VoxelVolume (mm)	5118.6±8540.8	1889.0±2466.1	7194.7±10384.6	<0.05	7194.7±10384.6	17365.0±19033.2	4698.5±5481.8	<0.05
**Types of Tumors**								
Adenosis	7	7	0	<0.05	8	8		0.125
Fibroepithelial	26	26	0		5	5	0	
Intraductal Lesion	6	6	0		4	4	0	
Intraductal Papilloma	6	6	0		3	3	0	
ICNST	42	0	42		10	0	10	
ICNST with DCIS	6	0	6		6	0	6	
ICNST with LCIS	7	0	7		5	0	5	
DCIS	10	0	10		4	0	4	

**Notes:** n represents the number of samples. ICNST refers to Invasive Carcinoma of Non-Special Type, ICNST with DCIS refers to Invasive Carcinoma of Non-Special Type with Ductal Carcinoma in Situ, ICNST with LCIS refers to Invasive Carcinoma of Non-Special Type with Lobular Carcinoma in Situ, and DCIS refers to Ductal Carcinoma in Situ. The *p*-value was calculated using the chi-square test.

**Table 2:** Inter-observer correlation coefficient (ICC) between observer (O) and standard (S).

**Observers**	**ICC value with 95% CI**						
	**Shape**	**First-Order**	**GLCM**	**GLDM**	**GLRLM**	**GLSZM**	**NGTDM**
O_1-S	0.978 (0.961,0.991)	0.987 (0.974, 0.994)	0.984 (0.968, 0.992)	0.978 (0.955, 0.989)	0.941 (0.880, 0.971)	0.956 (0.910, 0.978)	0.975 (0.951, 0.988)
O_2-S	0.985 (0.970, 0.993)	0.988 (0.976, 0.994)	0.986 (0.971, 0.993)	0.984 (0.968, 0.992)	0.977 (0.953, 0.989)	0.958 (0.916, 0.979)	0.982 (0.964, 0.991)
O_3-S	0.986 (0.971, 0.993)	0.990 (0.979, 0.995)	0.987 (0.973, 0.993)	0.987 (0.974, 0.994)	0.980 (0.959,0.991)	0.967 (0.932, 0.983)	0.987 (0.975, 0.993)
O_4-S	0.985 (0.970, 0.993)	0.987 (0.974, 0.994)	0.992 (0.984, 0.996)	0.990 (0.980, 0.995)	0.981 (0.961,0.991)	0.966 (0.930, 0.983)	0.985 (0.969, 0.992)
Average ICC	0.976 (0.963,0.988)	0.985 (0.975, 0.992)	0.983 (0.970, 0.991)	0.979 (0.965, 0.989)	0.959 (0.930,0.978)	0.945 (0.906, 0.970)	0.976 (0.958, 0.987)

**Table 3:** Comparison of performance analysis for models in differentiating between benign and malignant lesions.

**Model**	**Performance Metrics**						
	**AUC (Train)**	**AUC (Test)**	**CV AUC**	**Accuracy**	**Sensitivity**	**Specificity**	**F1 Score**
CatBoost	0.998 (0.989-1.000)	0.937 (0.852-0.993)	0.937 (+/- 0.122)	0.900	0.889	0.909	0.889
Random Forest	0.987 (0.968-0.999)	0.881 (0.758-0.972)	0.922 (+/- 0.129)	0.800	0.778	0.818	0.778
Naive Bayes	0.913 (0.849-0.962)	0.843 (0.707-0.949)	0.811 (+/- 0.141)	0.700	0.667	0.727	0.667
Logistic Regression	0.950 (0.903-0.985)	0.833 (0.693-0.947)	0.892 (+/- 0.147)	0.750	0.722	0.772	0.722
XGBoost	0.960 (0.919-0.991)	0.821 (0.677-0.949)	0.869 (+/- 0.237)	0.600	0.222	0.909	0.333
AdaBoost	0.958 (0.918-0.986)	0.814 (0.675-0.931)	0.870 (+/- 0.204)	0.700	0.611	0.772	0.648
k-NN	0.916 (0.8560-0.964)	0.813 (0.673-0.925)	0.785 (+/- 0.138)	0.750	0.611	0.864	0.688
SVC	0.770 (0.659-0.874)	0.763 (0.601-0.906)	0.727 (+/- 0.099)	0.600	0.944	0.318	0.680
MLP	0.688 (0.555-0.816)	0.662 (0.460-0.830)	0.715 (+/- 0.252)	0.600	0.111	1.000	0.200

## Data Availability

The datasets used in this study are private and not publicly available due to patient confidentiality and institutional restrictions.

## LIST OF ABBREVIATIONS

AdaBoost: Adaptive Boosting
AUC: Area Under Curve
CatBoost: Categorical Boosting
CI: Confidence Interval
DCE-MRI: Dynamic Contrast-Enhanced Magnetic Resonance Imaging
DWI: Diffusion-weighted Imaging
FOV: Field of View
GLCM: Gray Level Co-occurrence Matrices
GLDM: Gray Level Dependence Matrices
GLRLM: Gray Level Run Length Matrices
GLSZM: Gray Level Zone Size Matrices
ICC: Inter-Observer Correlation Coefficient
k-NN: k-Nearest Neighbors
LASSO: Absolute Shrinkage and Selection Operator
LightGBM: Light Gradient-Boosting Machine
ML: Machine Learning
MLP: Multi-layer Perceptron
NGTDM: Neighboring Gray Tone Difference Matrices
SHAP: SHapley Additive exPlanations
SVC: Support Vector Classifier
XGBoost: Extreme Gradient Boosting

## STANDARDS OF REPORTING

STROBE guidelines were followed
